# Mpox and Surgery: Protocols, Precautions, and Recommendations

**DOI:** 10.3390/microorganisms12091900

**Published:** 2024-09-15

**Authors:** Nikolaos Kamaratos-Sevdalis, Islam Kourampi, Nazli Begum Ozturk, Anna C. Mavromanoli, Christos Tsagkaris

**Affiliations:** 1 2nd Department of Internal Medicine, Tzaneio General Hospital of Piraeus, 185 36 Piraeus, Greece; kamaratosn@gmail.com; 2 Faculty of Medicine, National and Kapodistrian University of Athens, 157 72 Athens, Greece; 3 Department of Internal Medicine, Beaumont Health, Royal Oak, MI 48073, USA; nazlibegum.ozturk@corewellhealth.org; 4 European Student Think Tank, Public Health and Policy Working Group, 1058 Amsterdam, The Netherlands; amavroma@uni-mainz.de; 5 Faculty of Medicine, Aristotle University of Thessaloniki, 541 24 Thessaloniki, Greece

**Keywords:** monkeypox, Mpox, operating room, orthopoxvirus, Poxviridae infections, viral zoonoses, surgical procedures, operative

## Abstract

Mpox, also known as Monkeypox, is an infectious disease known to spread via direct contact and fomites, which poses a significant contagion risk in surgical settings and may increase the challenges already posed by COVID-19. Within the three years following the outbreak of Mpox, we conducted a review of the impact of Mpox on surgical practice. We searched Pubmed/Medline and Scopus, focusing on original studies and case reports in English or German. Our search terms included “Mpox”, “Monkeypox”, and “Surgery”. Out of 60 clinical or epidemiological studies, as well as expert opinions, brief reports, and pertinent literature reviews, eight were included after full-text assessment. We also incorporated two pertinent literature reviews, including a total of 10 papers, in this analysis. The main topics addressed by the literature are 1. manifestations of Mpox for surgical consideration or urgent management, for which it is important to consider whether a surgical approach is needed to address long-term Mpox-related lesions and 2. infection control in surgical settings, especially considering its impact on elective surgery and the well-being of healthcare workers. Mpox could affect surgical services and access to operating theaters. Unlike COVID-19, Mpox, compared to initial concerns, has not substantially compromised surgical delivery. However, limited reports exist on the surgical impact of Mpox. It is crucial to involve surgeons in Mpox diagnosis, educate surgical practitioners on its mimicry of common surgical conditions, enhance infection control during surgery, and ensure access to corrective surgery as a means of tackling the stigmatization associated with Mpox and sexually transmitted diseases in general.

## 1. Introduction

Mpox, also known as Monkeypox, is a viral illness caused by the Monkeypox virus, a DNA virus and a member of the Poxviridae family. Mpox is a zoonosis, originally endemic to Central and West Africa, most concentrated in the Democratic Republic of Congo (DRC) and traditionally thought to spread from animals to humans. The animal reservoir for the disease includes squirrels, rats, monkeys, primates, prairie dogs, hedgehogs, pigs, and mice found in the African regions [1].

Mpox’s first isolation and identification occurred in 1959 in monkeys during vaccine research. The first confirmed human case was a child in DRC in 1970, suspected to have smallpox [2]. So far, two genetically distinct clades have been identified. The Congo Basin (Central African) clade is more prevalent than the West African clade and has demonstrated human-to-human transmission capabilities, while the West African clade has not been reported to have demonstrated human-to-human transmission so far [3]. Additionally, the Central African clade has a higher fatality rate than the West African clade [4,5]. Most cases of Mpox occur in rural areas. It is suspected that underreporting may translate to an underestimation of the potential threat of this pathogen [1]. Males tend to be more susceptible to contagion in most African Mpox outbreaks. This is also supported by the fact that globally, 96.4% of cases are male, with a median age of 34 years [6]. A twofold explanation has been proposed in this regard; on one hand, androgenic hormones have a suppressing effect on immunity, and sex steroid hormones influence infection-resistance genes. On the other hand, cultural norms and traditions such as men engaging in farming, and in some communities, hunting activities, put the male population at greater risk [7].

Despite the fact that human-to-human transmission has previously been limited, in the context of decreasing herd immunity to orthopoxviruses, an increasing frequency of disease spread among humans has been reported [8]. Transmission may occur through close contact, fomites, exposure to large respiratory droplets, or during direct contact with the skin lesions of an infected person [9]. More specifically, analysis has shown that viral loads are high in bodily fluids, like saliva, urine, semen, and feces, as well as in oropharyngeal and rectal swabs, pointing to sexual transmission as a major driver of transmission. Judging by the lack of a clear epidemiological connection to West or Central Africa, the occurrence of Mpox in multiple regions raises questions about the possibility of long-term undiscovered transmission [6].

The Director-General of the World Health Organisation (WHO) declared the escalating global Mpox outbreak a Public Health Emergency of International Concern (PHEIC) on 23 July 2022 [10]. More than 120 countries reported Mpox cases between 1 January 2022 and 26 August 2024, with over 100,000 laboratory-confirmed cases. Mpox has been well documented in the DRC and Central Asia for over ten years, with a consistent rise in cases annually. Nevertheless, transmission was primarily observed from animals (rodents) to humans, although the potential of human-to-human transmission was recognized [11]. Previous research also acknowledged the limited ability of childhood vaccination against smallpox to prevent monkeypox infection [12]. Notably, 2022 and 2023 saw a notable spike in reported cases, and this year’s numbers have already surpassed last year’s total, with over 15,600 cases and 537 deaths recorded so far [13]. The threat should not be underestimated. The virus’s potential to mutate and the relaxation of safety measures make new outbreaks a real possibility. In particular, the eradication of smallpox leads to the cessation of smallpox vaccinations, which is believed to have contributed to the current Mpox outbreak. Cross-immunity, which occurs when immunity to one virus provides some level of protection against a related virus, is a key factor here. Since both Mpox and smallpox belong to the orthopoxvirus genus and share this cross-immunity, the decline in smallpox vaccination has reduced the protections against Mpox, facilitating its spread [14].

Surgery is rarely part of the discourse surrounding the management of infectious disease outbreaks. Nevertheless, the delivery of surgery can be compromised due to the spread of infections across surgical wards, affecting the well-being of the surgical workforce and patients, and requiring resources such as operating theaters and ventilators to be allocated to intensive care causes. As a matter of fact, the COVID-19 pandemic has had a profound impact on healthcare systems worldwide, leading to the postponement or cancellation of approximately 50% of non-urgent surgical procedures as part of infection control measures [15]. While the Mpox virus is a global concern in its own right, the pandemic has indirectly affected its management and spread. The disruption of routine healthcare services and the reallocation of resources to address COVID-19 have strained public health infrastructures, potentially compromising the surveillance and response capabilities for other infectious diseases, including Mpox.

Considering the aforementioned [5,6,10,14,15], our study aims to present evidence about the implications of Mpox on the delivery and practice of surgery, highlight high-quality practices, and identify potential knowledge gaps.

## 2. Materials and Methods

**Figure 1:**
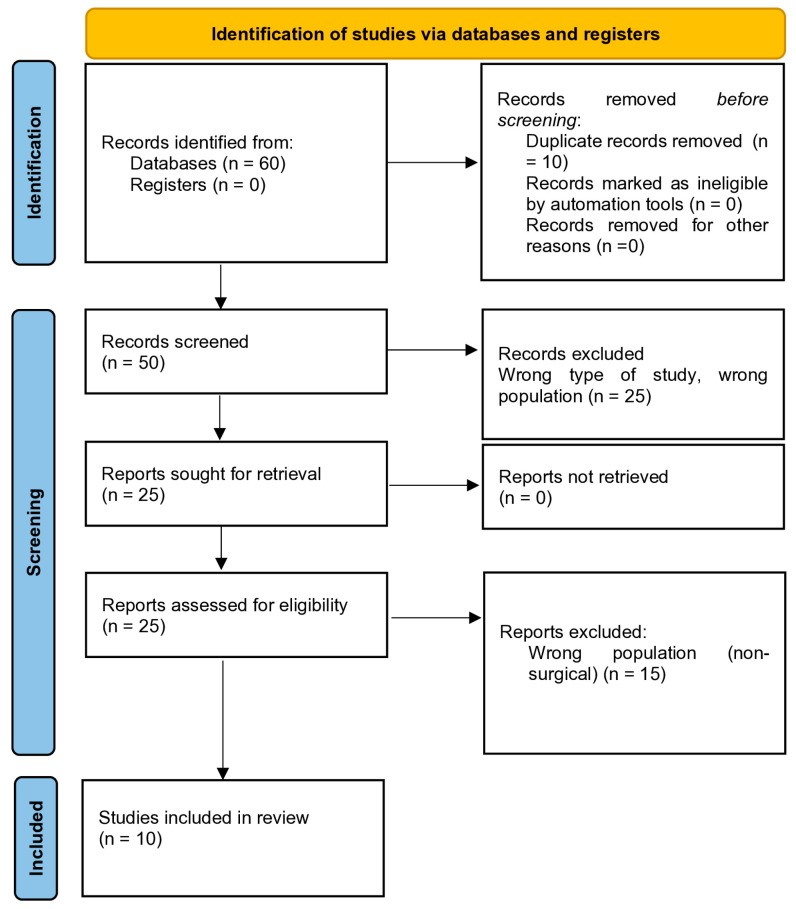
Mpox and surgery: protocols, precautions, and recommendations. Literature search and selection flowchart.

The literature search and selection were planned and executed by two independent researchers. Two major databases, namely Pubmed/Medline and Scopus, were searched from inception to 15 May 2024 for peer-reviewed articles addressing Mpox-associated surgical complications, risk of personnel contagion, and protective measures against Mpox in surgical settings. The search terms were a combination of terms relating to the disease (e.g., “Monkeypox” OR “Mpox”) and surgical practice (e.g., “surgery”). Additionally, reference lists and grey literature were examined in order to retrieve any relevant articles not found in our search. Original prospective or retrospective studies or case reports related to the above-mentioned search terms and themes, published from inception to 15 May 2024, were included. Given both the time constrictions and the instructive nature of infection control guidelines, short communications and expert opinions and reviews were also considered based on their relevance and publication in peer-reviewed outlets. Articles not available in English or German, the languages that the researchers involved in literature selection use in academic settings, as well as non-peer-reviewed papers, were not considered. The literature search and reporting were conducted according to the Preferred Reporting Items for Systematic Reviews and Meta-Analyses (PRISMA) checklist for scoping reviews (Figure 1) [16]. The quality of the studies included in the review was assessed using the Joanna Briggs Institute (JBI) critical appraisal checklists, tailored to each study design. The use of the JBI checklists ensured a consistent and thorough assessment of study quality. The detailed results of the quality assessment, including the completed JBI checklists for each study, can be found in Supplementary Material File S1 [17].

## 3. Results

**Figure 2:**
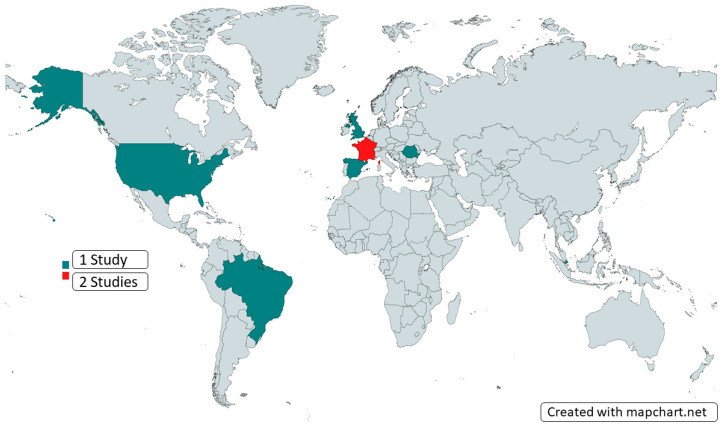
Countries of origin of the included studies.

Our comprehensive review encompassed 10 articles, namely 3 case reports, 2 literature reviews, 1 brief report, 1 observational study, and 3 expert recommendations. These studies reported the intersection of Mpox and surgery, based on data and knowledge gathered in 7 different countries across 3 continents (Figure 2). An overview of the studies, their methodology, and their outcomes is presented in Table 1.

The case reports pertained to patients aged between 42 and 48 years, co-diagnosed with HIV. Upon contracting Mpox, one became a kidney transplant recipient, one suffered from a peritonsillar abscess despite prior vaccination, and one suffered from odynophagia and a vesiculo-pustular rash affecting the facial, limb, inguinal, and scrotal regions [18,22,23].

The latter aligns with a short communication reflecting on the implications of future Mpox outbreaks in the frame of kidney transplantation surgery and postoperative care (Reis et al.). Accessing surgical care for Mpox patients, particularly those with compromised immune systems such as transplant recipients, poses significant challenges. Delays in diagnosis, stringent isolation protocols, and the need for specialized surgical teams may contribute to treatment challenges. To optimize outcomes for these patients, the integration of multidisciplinary teams comprising infectious disease specialists, transplant surgeons, and critical care experts is essential. This collaborative approach is important for navigating the intricate management of Mpox in surgical settings, ensuring both effective infection control and successful management of surgical complications [21,25,26].

The role of anesthesiologists and, subsequently, perioperative care in terms of mitigating the risk of Mpox spread in surgical settings was highlighted in two records [19,25,26]. Providing effective surgical care for patients diagnosed with Mpox or suspected of having monkeypox requires strict infection control measures in the perioperative period. This includes strict adherence to standard, contact, droplet, and airborne precautions due to the virus’s potential transmission through direct contact with lesions or respiratory secretions. Preoperative screening for symptoms such as fever, rash, and swollen lymph nodes is critical, and elective surgeries should be postponed until patients are deemed noninfectious, typically after symptoms or lesions have resolved. In facilities lacking airborne infection isolation rooms (AIIRs), it is recommended to implement air-cleaning technologies like high-efficiency particulate air (HEPA) filtration during and after aerosol-generating procedures. Effective environmental disinfection using US Environment Protection Agency (EPA)-registered products that target viral pathogens is essential to prevent nosocomial transmission.

In an observational study focusing on monkeypox proctitis, 42 out of 143 subjects who tested positive for Mpox were found to have Mpox proctitis [20]. This group, comprising only males with a median age of 39, frequently reported recent unprotected anal sex or new sexual partners. Cutaneous lesions were evident in half of these patients, primarily affecting areas such as the anogenital region, mouth, back, hands, and feet. Furthermore, a notable number of cases were also presented with oral or pharyngeal infections.

A risk-of-bias assessment was conducted with the JBI toolset (Supplementary Material File S1). Case reports authored by Attieh, Davido, and colleagues showed some risk of bias, particularly concerning unclear descriptions of patient demographics and takeaway lessons, while Oprea et al. demonstrated a lower risk overall [18,22,23]. Studies by Harvala, Teo, and colleagues had a low risk of bias, meeting all criteria positively [19,21].

The cohort study by Guevara-Martínez et al. exhibited a low risk of bias, though there were concerns regarding unclear reporting of follow-up time and management strategies [20]. Among expert opinions, Gouel-Cheron et al. and Tan et al. had a low risk of bias, while Reis et al. showed a higher risk associated with unclear source identification and standing in the field as per the JBI checklist [25].

## 4. Discussion

The 2022 Mpox outbreak presented a number of features that rendered it eligible for PHEIC status. Concerns raised over its rapid spread across continents and countries seemed to have reached surgical wards, prompting the publication of original, as well as secondary academic scholarship. While the former reported on Mpox as the cause of conditions often referred to or treated by surgeons or Mpox manifestations across surgical patients with immune deficits, the latter attempted to set the scene for providing safe surgical care in the event of further escalation of the outbreak.

Although the clinical reports and manifestations are unique to the pathogen and comparing them to manifestations of previous disease outbreaks such as COVID-19 or swine influenza would not benefit the discourse, preventive measures can be comparatively addressed. The measures put in effect to mitigate infectious outbreaks in surgical settings can usually be grouped under four categories, 1. use of PPE, 2. test and screening, 3. isolation, and 4. vaccination and immunization.

The use of PPE shares significant similarities with protocols used during previous non-Mpox infectious disease outbreaks; gowns and gloves as well as masks have been recommended in all cases. In the case of Mpox, minimizing physical contact has been a key aspect of PPE usage; therefore, less focus was bestowed on particular types of masks, such as conventional surgical masks and N95 masks. Testing of patients being admitted to surgical wards has also been instrumental. While the spread of COVID-19 prompted the development of rapid tests, the testing capacity for Mpox was derived mainly from polymerase chain reaction (PCR)-based analysis of samples collected from the lesions. The development of rapid-testing modalities providing a result within approximately 30 min has been reported; however, their rare use does not allow a comparison of their sensitivity and specificity to PCR. A preliminary analysis of a relevant product that received preliminary authorization in the USA yielded a sensitivity of 92%, although that was dependent on the origin of the sample; the latter could be oropharyngeal, rectal or cutaneous [28]. Isolation protocols were established for patients testing positive and transmitting the disease. While quarantine time for COVID-19 patients was adapted to clinical patterns of transmission across different mutations and was gradually reduced from 2 weeks to 5 days, Mpox isolation was rather PCR-compliant [29,30]. Evidence suggested isolation between 21 and 56 days for Mpox patients, representing the time needed until a negative sample could be obtained. The use of isolation rooms was recommended when procedures leading to the spreading of skin lesions, such as intubation, were performed [19]. Finally, yet importantly, although two vaccines against Mpox were available, there were no reports of a mandate of vaccination and subsequent differentiations in testing and isolation protocols with regard to inpatients in acute and elective settings [31].

On this basis, it appears that well-established protocols for infection control can be adapted to Mpox, in order to mitigate its spread in surgical settings. The following recommendations are drawn [25,32]:

- Testing needs to be performed at the time of admission to surgical departments. For patients undergoing surgery in acute settings, rapid test kits need to be used in parallel with conventional PCR testing.
- A positive test renders isolation for patients whose condition allows for the postponement of the operation.
- Patients tested positive for Mpox or considered positive on clinical grounds in the absence of a testing result (lesions, contact with Mpox-positive individuals) need to be operated on with Mpox-specific hygienic precautions. These include direct transfer from an isolation room to the operating theater and vice versa, consistent use of PPE by all parties involved, and a preference for local or regional over general anesthesia.
- An infection control checklist should be used at the point of the admission of the patient or before the entrance of the patient into the operating theater to ensure the appropriate documentation of all measures used and the implementation of additional preventive measures, if necessary and feasible.
- Mpox vaccination, as well as physical distancing practices across population groups and regions with a high rate of transmission, should be promoted, with a mandate being considered in the case of healthcare services, including elective operations.

These recommendations can be considered generic and particular features of their implementation need to be elaborated on in detail. This includes the regulation and availability of point-of-care testing modalities, the exact type of PPEs that are sufficient to prevent infection spread without affecting healthcare expenses or the ecological output of surgery, and the potential of Mpox vaccines to curb infection and transmission rates as an argument in favor or against a surgery-based vaccination mandate. The latter should also be stressed in light of recent studies reporting the spread of deadlier Mpox strains in Africa [33]. Providing equal and broad access to vaccination is instrumental to minimizing potential disruptions of surgical services. Additional measures proposed above can contribute to this goal, but cannot be considered sufficient to achieve unilaterally.

The present study is subject to a number of limitations that include the lack of a meta-analysis and the selective inclusion of both primary and secondary sources, such as short communications and reviews. With regard to bias, the studies ranged from a low to moderate risk of bias, with case reports and some expert opinions presenting the greatest concerns. A meta-analysis could not be performed due to the lack of comparable numerical outcomes in at least two of the included studies. The authors considered it also necessary to report and discuss secondary sources, given that evidence regarding infection control measures stemmed from these sources, rather than from clinical studies. Future research ought to tackle knowledge gaps with regard to the type, as well as the implementation of measures against Mpox, in surgical settings.

## 5. Conclusions

In conclusion, the 2022 Mpox outbreak underscores the need for infection control practices in surgical settings. Established methods, such as the use of personal protective equipment, testing and screening, isolation, and vaccination, which have been effective in previous infectious outbreaks, should be adopted for Mpox. Rapid testing at admission, isolation of positive cases, and specific hygienic precautions during surgeries are recommended. Promoting Mpox vaccination within the community has the potential to reduce disease transmission and decrease the incidence of postponing or performing surgery under infection control protocols. Future studies should aim to address existing knowledge gaps and appraise the implementation of Mpox control measures in surgical settings.

## Figures and Tables

**Table 1:** Tabular presentation of the included studies.

Title	Region	Study Type	Population	Results	Complications	Implications for Surgery	References
Disseminated Monkeypox Infection in a Kidney Transplant Recipient: A Case Report	USA	Case report	Kidney transplant recipient co-diagnosed with Mpox and HIV	Clinical manifestations of Mpox might be more severe in kidney transplant recipients	Urine retention and bowel obstruction	Conservative management	Attieh et al. [18]
What does the anesthesiologist need to know about monkeypox?	Singapore	Literature review	General population; healthcare providers; surgical patients	High transmission of Mpox outside endemic regions; antivirals may impact anesthesia	N/A	Anesthesiologists must recognize Mpox, use PPE properly, manage transfers, and perform risk stratification for staff exposure; need for protocols to minimize transmission	Teo et al. [19]
Proctitis in patients with monkeypox infection: a single-center analysis of 42 consecutive cases from a multidisciplinary observational study on monkeypox proctitis	Spain	Cross-sectional study	42 patients with monkeypox proctitis	Proctitis common among men who have sex with men (MSM); PCR essential for diagnosis; pain control key; early reevaluation advised	Proctitis, perianal abscess	Surgical drainage of an anal abscess was necessary in only three patients	Guevara-Martínez et al. [20]
Evaluating the risk of transfusion and transplant-transmitted monkeypox infections	UK	Literature review	Patients with Mpox	Discusses challenges in diagnosing Mpox infections, potential transmission through blood and organ transplants, and need for improved diagnostics	N/A	Importance of diagnostic measures to prevent potential transmission through surgical procedures and transplants	Harvala et al. [21]
Monkeypox outbreak 2022: an unusual case of peritonsillar abscess in a person previously vaccinated against smallpox	France	Case report	Patient with HIV, previously vaccinated	Mpox can present atypically with as deep tissue abscess	Peritonsillar abscess in a vaccinated individual	Awareness of potential unusual presentations that may require surgical evaluation	Davido et al. [22]
A severe case of mpox complicated with penile necrosis and keratitis in a patient living with HIV	Romania	Case report	Patient with HIV	Prolonged evolution and severe involvement of multiple body parts, emphasizing a multidisciplinary approach	Penile necrosis and keratitis	Surgical debridement was necessary for penile necrosis, showing severe cases may require surgical intervention alongside medical treatment	Oprea et al. [23]
Monkeypox-Associated Acute Kidney Injury and Foreseeable Impacts on Nephrology and Kidney Transplantation Services	Brazil	Editorial	Hospitalized patients with suspected or confirmed monkeypox infection	No clear evidence that patients with kidney transplant have worse outcomes with Mpox	Acute kidney injury	No clear surgical implications within the timeframe of the study	Reis et al. [24]
Monkeypox-infected patients in the perioperative context: Recommendations from an expert center	France	Expert recommendations	Patients with monkeypox infection in the perioperative context	Recommendations from an expert center	N/A	11 recommendations are being discussed	Gouel-Cheron et al. [25]
Perioperative Considerations for Patients with Mpox (Monkeypox)	Global	Expert recommendations	General population with confirmed Mpox infection, Healthcare providers, Perioperative patients	Importance of PPE if a clinical concern for Mpox.	Complications: secondary infections, bronchopneumonia, sepsis, encephalitis, corneal infection	: Defer elective surgery for Mpox patients until no risk of transmission Minimize equipment, limit OR traffic, use EPA-registered disinfectants	Tan et al. [26]
Transmission Precautions for Monkeypox Infection	Global (general guidelines)	Expert recommendations	Patients undergoing surgery with suspected or confirmed monkeypox infection	Postpone elective surgery if possible; minimize perioperative staff presence	N/A	-Postpone elective surgery if possible -Use comprehensive PPE. -Consider airborne precautions and isolation. -Ensure thorough cleaning and disinfection protocols	AORN [27]

Abbreviations: personal protective equipment (PPE), operating theatre (OR), US Environment Protection Agency (EPA), human immunodeficiency virus (HIV), Association of Perioperative Nurses (AORN).

## Data Availability

No primary data were generated in the frame of this study.

## Supplementary Materials

The following are available online at https://www.mdpi.com/article/10.3390/microorganisms12091900/s1, File S1: Risk of bias assessment.
