# Supplementary material for: Mpox and Surgery: Protocols, Precautions, and Recommendations
Source: Microorganisms. 2024 Sep 15;12(9):1900. doi: 10.3390/microorganisms12091900 (PMC11434558; doi:10.3390/microorganisms12091900)
Supplement: Supplementary file 1 [file microorganisms-12-01900-s001.zip › microorganisms-3169681-supplementary.pdf]

Supplementary Material: Risk of Bias Assessment

CASE REPORT:

	Q1	Q2	Q3	Q4	Q5	Q6	Q7	Q8
Attieh et al	No	yes	yes	yes	yes	yes	yes	unclear
Davido et al.	No	yes	yes	yes	yes	no	yes	yes
Oprea et al.	No	yes	yes	yes	yes	yes	yes	yes

Q1: Were patient's demographic characteristics clearly described?, Q2: Was the patient's history clearly described and presented as a timeline?, Q3: Was the current clinical condition of the patient on presentation clearly described?, Q4: Were diagnostic tests or assessment methods and the results clearly described?, Q5: Was the intervention(s) or treatment procedure(s) clearly described?, Q6: Was the post-intervention clinical condition clearly

described?, Q7: Were adverse events (harms) or unanticipated events identified and described?, Q8:Does the case report provide takeaway lessons?

NARRATIVE REVIEWS:

	Q1	Q2	Q3	Q4	Q5	Q6
Harvala et al.	Yes	yes	yes	yes	yes	yes
Teo et al	Yes	yes	yes	yes	yes	yes

Q1: Is the author or source of the narrative credible and appropriate? Q2: Is the relationship between the narrative and its context clearly explained (i.e., where, when, with whom, and how)? Q3: Does the narrative present events in a logical order that helps the reader understand how they unfold? Q4: Do you, as the reader, arrive at similar conclusions to those presented by the author? Q5: Do the conclusions follow logically from the narrative? Q6: Do you consider this account to be a well-constructed narrative?

CROSS SECTIONAL STUDY:

	Q1	Q2	Q3	Q4	Q5	Q6	Q7	Q8
Guevara-Martínez et al.	yes	yes	yes	yes	no	no	yes	unclear

Q1: Were the criteria for inclusion in the sample clearly defined?, Q2: Were the study subjects and the setting described in detail?,
Q3: Was the exposure measured in a valid and reliable way? Q4: Were objective, standard criteria used for measurement of the condition?,Q5: Were confounding factors identified?, Q6: Were strategies to deal with confounding factors stated?, Q7: Were the outcomes measured in a valid and reliable way?, Q8: Was appropriate statistical analysis used?

EXPERT OPINION:

	Q1	Q2	Q3	Q4	Q5	Q6
Gouel-Cheron et al.	yes	yes	yes	yes	yes	yes
Tan et al.	yes	yes	yes	yes	yes	yes
AORN.	unclear	yes	yes	yes	yes	n/a
Reis et al.	no	unclear	yes	yes	yes	n/a

Q1: Is the source of the opinion clearly identified?, Q2: Does the source of opinion have standing in the field of expertise?, Q3: Are the interests of the relevant population the central focus of the opinion?, Q4: Does the opinion demonstrate a logically defended argument to support the conclusions drawn?, Q5: Is there reference to the extant literature? Q6: Is any incongruence with the literature/sources logically defended?
